# Gender Differences and Psychopathological Features Associated With Addictive Behaviors in Adolescents

**DOI:** 10.3389/fpsyt.2017.00256

**Published:** 2017-12-01

**Authors:** Marco Di Nicola, Vittoria Rachele Ferri, Lorenzo Moccia, Isabella Panaccione, Annamaria Miriam Strangio, Daniela Tedeschi, Paolo Grandinetti, Antonino Callea, Fabio De-Giorgio, Giovanni Martinotti, Luigi Janiri

**Affiliations:** ^1^Institute of Psychiatry and Psychology, Fondazione Policlinico Universitario “A. Gemelli”, Università Cattolica del Sacro Cuore, Rome, Italy; ^2^NESMOS Department, School of Medicine and Psychology, Sant’Andrea Hospital, Sapienza University of Rome, Rome, Italy; ^3^Department of Human Science, LUMSA University, Rome, Italy; ^4^Section of Legal Medicine, Institute of Public Health, Università Cattolica del Sacro Cuore, Rome, Italy; ^5^Department of Neuroscience and Imaging, Institute of Psychiatry, “G. d’Annunzio” University of Chieti-Pescara, Chieti, Italy

**Keywords:** adolescence, substance use, gambling, Internet, impulsivity, dissociation, alexithymia, school performance

## Abstract

**Background:**

The aims of the study were to assess prevalence and gender differences of addictive behaviors (substance- and non-substance-related) in an adolescent population, and their association with psychopathological features and academic performance.

**Material and methods:**

A sample of high school Italian students (*n* = 996; M = 240, F = 756) was examined using a self-report survey concerning sociodemographic characteristics, cigarette smoking, alcohol and substance use, perceived academic performance, activities, and behaviors (Internet use, gambling, and physical exercising). The Internet Addiction Test, the South Oaks Gambling Screen-revised Adolescent, and the Exercise Addiction Inventory-Short Form were administered to identify problematic behaviors. The Barratt Impulsiveness Scale for Adolescent, the Snaith–Hamilton Pleasure Scale, the Dissociative Experience Scale for Adolescent, and the Toronto Alexithymia Scale were used to investigate psychopathological dimensions.

**Results:**

Frequent alcohol intake and lifetime substances consumption were more common among males. The occurrence of other addictive behaviors was 22.1% for problematic Internet use (M = F), 9.7% for at-risk/problematic gambling (M > F), and 6.2% for maladaptive physical exercise (M = F). We also found an association between substance-/non-substance-related addictive behaviors and psychopathological dimensions. Addictive behaviors were more frequent among students reporting poor school performance.

**Conclusion:**

Our study showed a relevant prevalence of addictive behaviors in a sample of Italian high school students, with specific gender differences. We underlined the cooccurrence of substance and non-substance-related addictive behaviors, and their association with worse school performance. Dissociative proneness, anhedonia, alexithymia, and impulsivity were associated with addictive behaviors in adolescents and might represent vulnerability factors for the development of psychiatric disorders in adulthood. A better understanding of psychopathological features associated with addictive behaviors might be useful for the prevention/early intervention.

## Introduction

Adolescence has far been recognized as a critical developmental period for several reasons, including dramatic physical, cognitive, and psychosocial changes occurring at that time (1, 2). Both substance- and non-substance-related addictive behaviors usually onset in adolescence or young adult age and are more prevalent in these age groups than in any others (3). According to the emerging neurobiological model of addiction, neurodevelopmental changes occurring during adolescence lead to an imbalance between emotional (reward motivation) and cognitive processes (executive control) (3, 4). In fact, while the limbic system undergoes remarkable remodeling during puberty, prefrontal areas development is not complete until near the age of 25 (5). This neurobiological fragility may contribute in adolescence to a higher risk of developing addictive behaviors (6).

An individual susceptibility constituted by genetic, physiological, and personality characteristics may predispose adolescents to addictive tendencies. However, environmental factors, including early exposure to traumatic life events, familiar history of addictive disorders, increased accessibility to gambling and substances of abuse, and peer influence, have been recognized as risk factors as well (7, 8).

The last European School Survey Project on Alcohol and other Drugs (ESPAD) report has extended the scope of the survey to include not only novel substances of abuse but also behavioral addictions, such as problematic Internet use, gaming, and gambling (9). The ESPAD group points out that “the development of patterns of addictive Internet use among children and adolescents needs to be closely monitored and investigated” and that “measures to prevent adolescents from developing problems associated with gambling, such as debt, psychological deficits and social disadvantages, are of high priority.”

Distinct psychopathological dimensions have been consistently correlated to the occurrence of addictive disorders in adults (10–13). In the past several years, a growing body of research has started to unravel the complex interaction that occurs between psychopathology and the vulnerability to both substance and non-substance addictive behaviors in adolescents (14–20). Psychopathological factors, including both internalizing and externalizing symptomatology, have been further conceptualized in terms of both predictors and consequences of addictive tendencies in adolescents (19, 21, 22).

However, there is still limited evidence about occurrence, related features, and impact of addictive behaviors in this population. Therefore, the aims of the study were to assess prevalence, gender differences, psychopathological features, and academic performance associated with addictive behaviors (substance- and non-substance-related) in high school subjects.

## Materials and Methods

### Procedure

**Figure 1:**
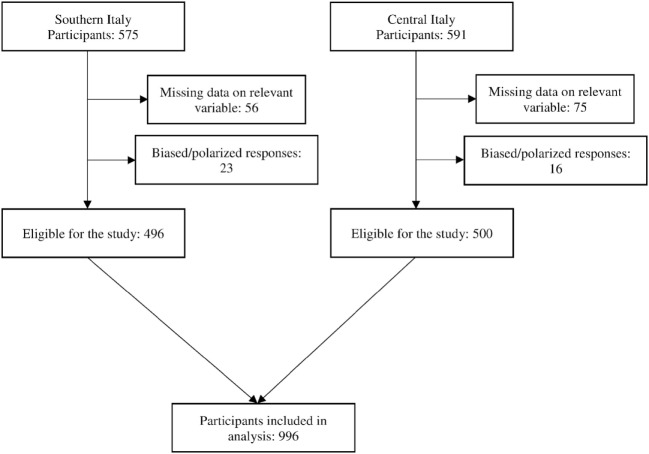
Study flow chart.

This cross-sectional study was conducted between 2014 and 2016 in central and southern Italy. From the initial sample of 1,174 students, 178 were not admitted to the study due to missing data on important variables or response polarization. The final sample consisted of 996 subjects (M = 240, F = 756) (Figure 1).

Anonymity was guaranteed to all participants. The study was approved by the Ethics Committee of Università Cattolica del Sacro Cuore, Rome. It was conducted in accordance with Good Clinical Practice guidelines and the Declaration of Helsinki (1964) and subsequent revisions. All subjects enrolled (or parents/tutors, if they were underage) gave their written informed consent before their inclusion in the study and participated without receiving any form of payment.

We used a self-report survey assessing sociodemographic characteristics, school grade and performance, parents’ marital status and occupation, cigarette smoking, alcohol and substances consumption, and non-substance-related addictive behaviors such as Internet using, gambling, and physical exercising.

To screen for non-substance-related at-risk/problematic behaviors, we administered the Italian versions of:

– Internet Addiction Test [IAT; (23, 24)]. It consists of 20 items evaluating the impact of Internet use in daily life. Items are rated in a five-point Likert scale (from 1—not at all—to 5—always). The total score distinguishes: average users with a full control of their usage (20–49), subjects with frequent problems because of excessive Internet use (50–79), or having significant problems because of Internet use (80–100).
– South Oaks Gambling Screen-revised Adolescent [SOGS-rA; (25, 26)]. It is a screening questionnaire for problematic behaviors associated with gambling. The scale refers to the 12 months before the survey and is composed of 12 items investigating the loss of control on the game, the run-up to the losses, interference with daily life, and feelings of guilt related to the game. Scores are used to define three categories of players: no problem (0–1), at-risk (2–3), and problematic (≥4).
– Exercise Addiction Inventory-Short Form [EAI-SF; (27)]. It consists of six statements rated on a 5-point Likert scale (from 1—strongly disagree—to 5—strongly agree). Total scores identify three categories of individuals: asymptomatic (0–12), symptomatic (13–23), and at-risk for exercise addiction (≥24).

To assess psychopathological dimensions, the Italian version of the following scales was employed:

– Barratt Impulsiveness Scale, Adolescent Version [BIS-11-A; (28)]. It is a 30-item self-reported questionnaire that targets impulsivity in adolescents. As to authors’ recommendations, a single factor model of the scale, including only BIS-11-A total score, was adopted.
– Snaith–Hamilton Pleasure Scale [SHAPS; (29, 30)]. It is a 14-item self-rating scale exploring hedonic responses in common pleasurable situations related to leisure pursuit and interests, eating and drinking, social interactions, and sensory experiences. Previous findings support the use of SHAPS for assessing anhedonia in adolescent populations (31).
– Dissociative Experience Scale for Adolescence [A-DES; (32, 33)]. It is a self-report screening questionnaire assessing dissociative symptoms in adolescents. It includes four subscales, namely, dissociative amnesia; depersonalization and derealization; absorption and imaginative involvement; and passive influence. A-DES proved to be a valid instrument, with very good internal reliability (34).
– Toronto Alexithymia Scale [TAS-20; (35, 36)]. It is a 20-item self-report instrument that assesses alexithymia. It has a three-factor structure, which includes the following subscales: difficulty identifying feelings; difficulty describing feelings; and externally oriented thinking. TAS-20 demonstrated good psychometric properties in adolescent populations (37).

### Statistical Analysis

The statistical package SPSS 21.0 for Mac (SPSS Inc., Chicago, IL, USA) was used for all the analyses. Dichotomous data were compared by chi-square test. Ordinal variables were compared by non-parametric Mann–Whitney *U* test. Continuous data were expressed as means ± SD and compared by independent Student’s *t*-test. Spearman’s rank correlation coefficient was employed to examine the relationship between continuous and ordinal variables. Logistic and linear regressions, through multiple regression method, were performed to identify the association of psychopathological variables with substance-/non-substance-related addictive behaviors. The statistical significance was set at *p* < 0.05.

## Results

### Participants and Sociodemographic Characteristics

Sociodemographic data are reported in Table 1. No differences between male and female students were found, except for parent’s marital status (χ^2^ = 8.337, *p* < 0.05; in males, the percentage of “married” and “separated/divorced” was higher and lower than females, respectively), mother’s work (χ^2^ = 19.300, *p* < 0.05; in males, the percentage of “staff employed” and “deceased/absent/does not know” was higher, while the percentage of “precarious,” “housewife,” and “entrepreneur” was lower than females), and school performance (χ^2^ = 17.998; *p* < 0.01; the percentage of “failure” and “poor” was higher for males, while the percentage of “satisfactory,” “good,” and “excellent” was higher for females).

**Table 1:** Sociodemographic characteristics.

	Total	Male	Female
*N*	996	240 (24.1)	756 (75.9)
Age (M ± SD)	16.47 ± 4.85	16.5 ± 5.61	16.47 ± 4.58

**Parent’s marital status**			
Married	336 (67.9)	97 (75.2)	239 (65.3)
Separated/divorced	124 (25.1)	22 (17.1)	102 (27.9)
Other	35 (7)	10 (7.7)	25 (6.8)

**Father’s work**			
Unemployed	4 (0.8)	2 (1.6)	2 (0.6)
Precarious	8 (1.7)	2 (1.6)	6 (1.7)
Staff employed	180 (37.7)	46 (36.2)	134 (38.2)
Professional employee	203 (42.4)	53 (41.7)	150 (42.7)
Stay at-home dad	1 (0.2)	1 (0.8)	0 (0)
Pensioner	10 (2.1)	2 (1.6)	8 (2.3)
Entrepreneur	52 (10.9)	13 (10.2)	39 (11.1)
Deceased/absent/does not know	20 (4.2)	8 (6.3)	12 (3.4)

**Mother’s work**			
Unemployed	14 (2.9)	4 (3.2)	10 (2.8)
Precarious	17 (3.6)	2 (1.6)	15 (4.3)
Staff employed	194 (40.6)	57 (45.6)	137 (38.8)
Professional employee	141 (29.6)	39 (31.2)	102 (29)
Housewife	68 (14.3)	10 (8)	58 (16.5)
Pensioner	3 (0.6)	0 (0)	3 (0.9)
Entrepreneur	17 (3.6)	2 (1.6)	15 (4.3)
Deceased/absent/does not know	23 (4.8)	11 (8.8)	12 (3.4)

**School class**			
1st	130 (13.1)	37 (15.5)	93 (12.4)
2nd	85 (8.6)	21 (8.8)	64 (8.5)
3rd	296 (29.9)	69 (28.9)	227 (30.2)
4th	234 (23.6)	56 (23.4)	178 (23.7)
5th	245 (24.8)	56 (23.4)	189 (25.2)

**School performance**			
Failure	95 (9.6)	32 (13.4)	63 (8.4)
Poor	193 (19.5)	63 (26.3)	130 (17.4)
Satisfactory	342 (34.6)	72 (30.1)	270 (36)
Good	293 (29.7)	58 (24.3)	235 (31.4)
Excellent	65 (6.6)	14 (5.9)	51 (6.8)

*The relative percentages are shown in brackets*.

*N, number of study participants; M, mean; SD, standard deviation*.

### Substance-Related Addictive Behaviors

Smoking habit, alcohol intake, and related patterns are described in Table 2. Prevalence of current smoking was 51.1%, with no significant gender differences. Prevalence of current alcohol use was 84.7%; 57.6% (M = 64.1%; F = 55.6%) of the students reported drinking at least once per month, while 30.8% (M = 40%; F = 27.9%) stated taking alcohol once a week or more, with significant gender differences. Frequency of drunkenness episodes was significantly higher in males. No gender differences were found on binge drinking, defined as consuming ≥6 drinks on a single occasion during the past month (38).

**Table 2:** Cigarette smoking, alcohol intake, and related patterns.

	Total	Male	Female	Comparison between groups
**Cigarette smoking**				
No, never	485 (48.9)	126 (52.9)	359 (47.6)	*U* = 84,935
Yes, sometimes	190 (19.2)	39 (16.4)	151 (20.1)	*p* = 0.160
Yes, often	58 (5.9)	18 (7.6)	40 (5.3)	
Yes, everyday	258 (26)	55 (23.1)	203 (27)	

**Alcohol intake**				
Never	151 (15.3)	36 (15.2)	115 (15.3)	*U* = 76,222.5
Less than once per month	268 (27.1)	49 (20.7)	219 (29.1)	*p* < 0.01
Once per month	169 (17.1)	31 (13.1)	138 (18.4)	
Several times a month	96 (9.7)	26 (11)	70 (9.3)	
Once a week	169 (17.1)	42 (17.7)	127 (16.8)	
Several times a week	109 (11)	40 (16.8)	69 (9.2)	
Everyday	27 (2.7)	13 (5.5)	14 (1.9)	

**Drunkenness**				
Never	287 (35.1)	60 (30.6)	227 (36.6)	*U* = 58,513.5
One time	168 (20.6)	36 (18.4)	132 (21.3)	*p* < 0.05
Sometimes	226 (27.7)	60 (30.6)	166 (26.8)	
Often	109 (13.4)	29 (14.8)	80 (12.9)	
Always	26 (3.2)	11 (5.6)	15 (2.4)	

**Binge drinking**				
No	745 (90.8)	174 (88.3)	571 (91.5)	χ^2^ = 1.61
Yes	76 (9.2)	23 (11.7)	53 (8.5)	*p* = 0.209

*The relative percentages are shown in brackets*.

*U, Wilcoxon–Mann–Whitney test; χ^2^, chi-square test; p, p value (statistical significance)*.

Illicit substances consumption and related patterns are reported in Table 3. Comparing the two groups, we found a higher frequency of lifetime illicit substances consumption and multiple substance use in males. Cannabis was the most used drug; 304 subjects (84.8% of substance users) consumed cannabis alone at least once in lifetime and 15 of them (4.8%) reported a daily consumption. Cannabis use was more frequent among females.

**Table 3:** Illicit substances consumption and related patterns.

	Total	Male	Female	Comparison between groups
**Lifetime substances consumption**				
No	632 (63.6)	131 (55)	501 (66.3)	χ^2^ = 9.855
Yes	362 (36.4)	107 (45)	255 (33.7)	*p* < 0.01

**Types of substances**				
Cannabinoids	304 (84.8)	77 (73.3)	227 (89.7)	χ^2^ = 20.394
Stimulants	2 (0.6)	0 (0)	2 (0.8)	*p* < 0.001
Other	2 (0.6)	2 (1.9)	0 (0)	
More than one	50 (14)	26 (24.8)	24 (9.5)	

**Frequency of use**				
Less than once per month	144 (45.8)	31 (32.6)	113 (51.6)	*U* = 8,048
Once per month	27 (8.6)	7 (7.4)	20 (9.1)	*p* < 0.001
Several times a month	42 (13.4)	14 (14.7)	28 (12.8)	
Once a week	34 (10.8)	11 (11.6)	23 (10.5)	
Several times a week	52 (16.6)	26 (27.4)	26 (11.9)	
Everyday	15 (4.8)	6 (6.3)	9 (4.1)	

*The relative percentages are shown in brackets*.

*U, Wilcoxon–Mann–Whitney test; χ^2^, chi-square test; p, p value (statistical significance)*.

### Non-Substance-Related Addictive Behaviors

Non-substance addictive behaviors and related patterns are reported in Table 4, while IAT, SOGS-rA, and EAI-SF scores are shown in Table 5.

**Table 4:** Non-substance addictive behaviors and related patterns.

	Total	Male	Female	Comparison between groups
**Time online**				
<1 h	169 (19)	47 (21.7)	122 (18.1)	*U* = 81,268.5
1–2 h	373 (41.8)	101 (46.5)	272 (40.4)	*p* < 0.05
3–4 h	196 (22)	45 (20.7)	151 (22.4)	
5–6 h	80 (9)	11 (5.2)	69 (10.2)	
<8 h	28 (3.1)	4 (1.8)	24 (3.6)	
>8 h	45 (5.1)	9 (4.1)	36 (5.3)	

**Gambling online**				
Never	765 (86.7)	153 (71.9)	612 (91.6)	*U* = 56,791.5
One time	43 (4.9)	16 (7.5)	27 (4)	*p* < 0.001
Sometimes	46 (5.2)	25 (11.7)	21 (3.1)	
Often	15 (1.7)	9 (4.2)	6 (0.9)	
Always	13 (1.5)	10 (4.7)	3 (0.4)	

**Gambling**				
Never	589 (65.7)	74 (34.1)	515 (75.8)	*U* = 57,258
One time	69 (7.7)	24 (11.1)	45 (6.6)	*p* < 0.001
Sometimes	164 (18.3)	65 (30)	99 (14.7)	
Often	50 (5.6)	37 (17.1)	13 (1.9)	
Always	24 (2.7)	17 (7.7)	7 (1)	

*The relative percentages are shown in brackets*.

*U, Wilcoxon–Mann–Whitney test; p, p value (statistical significance)*.

**Table 5:** Gender differences on IAT, SOGS-rA, and EAI-SF scores.

	Total	Male	Female	Comparison between groups
**IAT scores** (M ± SD)	40.93 ± 13.40	41.26 ± 13.64	40.83 ± 13.34	*t* = 0.416
				*p* = 0.678
**IAT results**				
Average users	776 (77.9)	179 (74.3)	597 (78.9)	*U* = 79,777
Frequent problems	212 (21.3)	61 (25.7)	151 (20)	*p* = 0.199
Significant problems	8 (0.8)	0 (0)	8 (1.1)	
**SOGS-rA scores** (M ± SD)	0.46 ± 1.40	1.35 ± 2.21	0.19 ± 0.89	*t* = 11.478
				*p* < 0.001
**SOGS-rA results**				
No problem	858 (90.3)	155 (70.1)	703 (96.3)	*U* = 59,402.5
At-risk	47 (4.9)	30 (13.6)	17 (2.3)	*p* < 0.001
Problematic	46 (4.8)	36 (16.3)	10 (1.4)	
**EAI-SF scores** (M ± SD)	13.18 ± 5.71	14.53 ± 5.52	12.76 ± 5.71	*t* = 4.151
				*p* < 0.001
**EAI-SF results**				
Asymptomatic individual	509 (52.4)	91 (39.1)	418 (56.6)	*U* = 71,654
Symptomatic individual	402 (41.4)	127 (54.5)	275 (37.3)	*p* < 0.001
At-risk for exercise addiction	60 (6.2)	15 (6.4)	45 (6.1)	

*The relative percentages are shown in brackets*.

*IAT, Internet Addiction Test; SOGS-rA, South Oaks Gambling Screen-revised Adolescent; EAI-SF, Exercise Addiction Inventory-Short Form; M, mean; SD, standard deviation; U, Wilcoxon–Mann–Whitney test; t, Student’s t-test; p, p value (statistical significance)*.

8.2% of students spent on the Internet more than 6 h/day; 220 subjects (22.1%) showed a maladaptive Internet use, according to IAT cutoff (“frequent” and “significant problems”). We found a significant correlation between IAT score and time spent online/day (rho = 0.381; *p* < 0.01), but no gender differences.

8.3% of the sample (*n* = 74) gambled “often” or “always,” with higher prevalence among males (M = 24.8%; F = 2.9%). Overall, 9.7% of the sample (29.8% of gamblers) showed a maladaptive gambling behavior, according to SOGS-rA cutoff (“at-risk/problematic” subjects), with significant gender differences (M = 29.9%; F = 3.7%). Moreover, a correlation between frequency of gambling and risk of related problematic behaviors (rho = 0.398; *p* < 0.01) was noted. Online gambling was apparently less frequent, with only 3.2% of the sample reporting having gambled online “often” or “always” with relevant gender differences (M = 8.9%; F = 1.3%).

The prevalence of maladaptive physical exercise (EAI-SF cutoff) was 6.2%, with no gender differences.

Finally, we found correlations between IAT, SOGS-rA, and EAI-SF scores (IAT*SOGS-rA: rho = 0.174; *p* < 0.01; IAT*EAI-SF: rho = 0.067; *p* < 0.05; SOGS-rA*EAI-SF: rho = 0.166; *p* < 0.01). SOGS-rA scores were also correlated to the frequency of alcohol use (rho = 0.200; *p* < 0.01) and cigarette smoking (rho = 0.084; *p* < 0.01).

### Psychopathological Features Associated With Gender and Addictive Behaviors

Significant gender differences on psychopathological variables are presented in Table 6. The TAS-20 “Difficulty describing feelings,” “Difficulty identifying feelings,” and total scores were higher among females.

**Table 6:** Gender differences on psychopathological features.

	Total	Male	Female	Comparison between groups
**TAS-20** total score	51.72 ± 11.53	49.37 ± 12.11	52.43 ± 11.26	*t* = −3.485; *p* < 0.01
Difficulty describing feelings	16.28 ± 6.1	14.61 ± 6.03	16.79 ± 6.04	*t* = 4.711; *p* < 0.001
Difficulty identifying feelings	15.5 ± 6.54	13.68 ± 5.73	16.05 ± 6.67	*t* = −4.773; *p* < 0.001
Externally oriented thinking	19.98 ± 5.49	20.86 ± 5.1	19.72 ± 5.61	*t* = −2.737; *p* < 0.01
**A-DES** total score	1.99 ± 1.14	1.29 ± 1.26	2.23 ± 1.07	*t* = −2.197; *p* < 0.05
Dissociative amnesia	1.85 ± 1.07	1.83 ± 1.28	1.86 ± 0.99	*t* = −0.232; *p* = 0.817
Absorption and imaginative involvement	2.69 ± 0.19	2.09 ± 2.20	2.91 ± 2.3	*t* = −3.515; *p* < 0.001
Depersonalization and derealization	1.61 ± 0.92	1.48 ± 0.93	1.66 ± 0.91	*t* = −1.976; *p* = 0.059
Passive influence	1.95 ± 1.16	1.92 ± 1.17	1.99 ± 1.15	*t* = −2.654; *p* = 0.061
**SHAPS** total score	1.23 ± 1.79	1.64 ± 2.20	1.10 ± 1.62	*t* = 3.986; *p* < 0.001
**BIS-11-A** total score	64.09 ± 9.99	63.81 ± 11.41	64.09 ± 9.99	*t* = −0.493; *p* = 0.622

*Values are expressed as mean ± standard deviation (M ± SD)*.

*TAS-20, Toronto Alexithymia Scale; A-DES, Dissociative Experience Scale for Adolescence; SHAPS, Snaith–Hamilton Pleasure Scale; BIS-11-A, Barratt Impulsiveness Scale for Adolescents; t, Student’s t-test; p, p value*.

The A-DES “Absorption and Imaginative Involvement” factor and total scores were higher in females. The A-DES “Dissociative Amnesia” factor score was higher in binge drinkers (*t* = −3.228; *p* < 0.01) and was associated with alcohol consumption (rho = 0.232; *p* < 0.01) and drunkenness episodes (rho = 0.223; *p* < 0.01).

No gender difference on BIS-11-A total score was observed. Binge drinkers obtained higher BIS-11-A total scores (*t* = −4.325; *p* < 0.001). BIS-11-A total score was also associated with drunkenness episodes (rho = 0.252; *p* < 0.01) and cigarette smoking (rho = 0.267; *p* < 0.01).

Logistic regression analysis showed that binge drinking was associated with TAS-20 “Difficulty Identifying Feelings” (*b* = 1.072; *p* < 0.001) and “Difficulty Describing Feelings” factors (*b* = 0.945; *p* < 0.01), with A-DES “Dissociative Amnesia” factor (*b* = 1.107; *p* < 0.001) and total scores (*b* = 0.986; *p* < 0.001), and with BIS-11-A total score (*b* = 1.036; *p* < 0.001).

Multiple regression analysis also highlighted that problematic Internet use (as to IAT) was related to both A-DES “Absorption and Imaginative Involvement” factor (*b* = 0.323; *p* < 0.001) and total scores (*b* = 0.111; *p* < 0.05), and with TAS-20 (*b* = 0.193; *p* < 0.001) and BIS-11-A (*b* = 0.236; *p* < 0.001) total scores (*R*^2^ = 0.176). Maladaptive gambling behavior (as to SOGS-rA) was associated with SHAPS total (*b* = 0.182; *p* < 0.001) and A-DES “Dissociative Amnesia” factor (*b* = 0.279; *p* < 0.001) scores (*R*^2^ = 0.054). Problematic physical exercise was associated with TAS-20 (*b* = 0.149; *p* < 0.01), A-DES (*b* = 0.155; *p* < 0.01) and, inversely, with BIS-11-A (*b* = −0.209; *p* < 0.001) total scores (*R*^2^ = 0.064).

### Academic Performance

40.9% (*n* = 148) of students with lifetime history of substance consumption (*n* = 362) reported failure/poor academic performance compared to 23.4% (*n* = 148) of subjects without it (*n* = 632) (χ^2^ = 38.31; *p* < 0.001). Negative correlations between perceived school performance, maladaptive Internet use (IAT total score; rho = −0.208; *p* < 0.01), and impulsivity traits (BIS-11-A total score; rho = −0.335; *p* < 0.01) were also found.

## Discussion

Our findings confirm a relevant prevalence of substance- and non-substance addictive behaviors among high school Italian students. In line with previous studies, the percentage of subjects who reported drinking several times a week/everyday was 13.7%, with a predominance of males (9, 39, 40).

Worryingly, our data revealed that 9.2% of students experimented binge drinking as a usual pattern of alcohol consumption, with no gender differences. A similar scenario emerged from the ESPAD study, in which 13% of the students reported “intoxication in the last 30 days,” with a slight preponderance of males (9).

Due to its cognitive and psychomotor effects on reaction time and coordination (41), alcohol use significantly contributes to the incidence of injuries, accidents, and other traumas, particularly among younger age groups. For alcohol-related injuries, binge drinking has been found to be a major factor (42).

Adolescence is a critical period for brain development, and the adolescent brain is particularly sensitive to the effect of alcohol and other psychoactive substances. Studies suggest that the specific consumption pattern of alternating alcohol intoxications and abstinent episodes, which is linked to excitotoxic cell death during withdrawal, may be deleterious for the nervous system (43). Binge drinking may therefore result in long-term changes in brain functioning.

Binge drinking is also associated with both acute (i.e., hangovers, blackouts, memory loss, etc.) and long-term clinical consequences, including the progression to established alcohol use disorders (44). Furthermore, binge drinking may lead to unprotected sexual activity or sexual assaults (45).

In our study, the use of both alcohol and illicit drugs coexisted in 33.6% of participants and, consistently with previous reports, was more common among males (9, 39, 40). The prevalence of substance use was higher than described by ESPAD (36.4 vs. 28%) (9), with cannabinoids being the most frequently consumed substances (84.8%). In contrast to previous findings, in our sample lifetime cannabinoids consumption was higher among females (46). As recently pointed out, gender difference in cannabis use has decreased over time, and this gap is progressively narrowing (47). Worryingly, studies on effects of cannabinoid exposure during adolescence in both humans and preclinical models suggest that females are more vulnerable to be deleteriously affected by these substances (48).

Few data are still available on the prevalence of non-substance addictive behaviors among adolescents. 22.1% of study subjects showed a maladaptive Internet use that was related to the number of hours/day spent online rather than the number of days/week (49). In our sample, the prevalence of subjects with problematic use of Internet is higher than reported by previous European studies (50, 51). This finding might be related to the increase of Internet use over the years and to progressively younger age of children currently accessing the Web (52).

We found that 9.7% of students were at risk for problematic gambling, showing a correlation with the frequency of gambling. The prevalence of problematic gambling was higher than observed in other European countries (53, 54).

The occurrence of maladaptive physical exercise was 6.2%. A research conducted among young adults by Meulemans and colleagues (55) stated that 3.3% of students were characterized as “at-risk” for exercise addiction, and 51.5% as “non-dependent-symptomatic,” while another study reported a prevalence of exercise addiction ranging from 23.8 to 26.2% (56).

No gender differences were found for problematic Internet use (M = 25.7%; F = 21.1%) and physical exercise (M = 6.4%; F = 6.1%), while problematic gambling, as previously observed in other studies, was significantly more common in males (M = 29.9%; F = 3.7%) (9, 57, 58).

Interestingly, problematic Internet use, gambling, and physical exercise were related with the frequency of alcohol consumption. The cooccurrence of alcohol/substances use and addictive behaviors, such as Internet use and gambling, have been already observed in adults (59, 60) and adolescents (56, 61–63). This cooccurrence may be explained by common biological factors and/or personality traits as impulsivity and sensation/novelty seeking (64).

Specific psychopathological features might also contribute to the development of distinct addictive behaviors, and possibly to an increased proneness for multiple cooccurring addictions (59). Accordingly, in our study, dissociative liability, anhedonia, alexithymia, and impulsivity levels have been found to be associated with the occurrence of addictive behaviors in adolescents. Of note, we observed that dissociative symptoms seem to be a common feature across diverse types of maladaptive behaviors (i.e., binge drinking, problematic gambling, Internet use, and physical exercise) consistently with previous reports (65–67). Dissociation is usually conceptualized as a lack of the integrative functions of memory, consciousness, and identity and is often related to traumatic experiences. However, non-pathological dissociative symptoms are quite common in the general population and several studies suggest that dissociation cannot be simply considered as a learned strategy to decrease emotional engagement (68), but it can be rather regarded as a structural, sometimes dysfunctional, emotion-regulation strategy (69, 70). In fact, dissociative proneness is related to deficits in the ability to symbolize and mentalize affective experiences (71), and elevated levels of dissociation are related to cognitive impairments in healthy subjects (72).

Taken together, the propensity to experience dissociative states, the inability to express and understand self-affects, the tendency to act rapidly and without reflexive thinking, along with a blunted capacity of experiencing pleasure in everyday life may thus be thought as vulnerability factors for addictive behaviors in adolescence. These psychopathological features may reflect a more general individual incapacity to regulate [i.e., to mentalize; (73)] affects induced by stressful events which ultimately leads to repetitive, dysfunctional behavioral patterns (74, 75). Consistently with theories that recognize an influence of addictive behaviors on cognitive and affective capacities of adolescents, both substance and behavioral addictions have been recently conceptualized as specific forms of maladaptive self-regulatory strategies (76, 77). In line with a unifying psychological perspective of addictions, the particularly intense and rigid relationship between the individual and his substance, or activity of choice, contributes to the development of the addictive process (78).

Finally, as shown in our and previous studies, specific psychopathological features seem to play a significant role in the development of both substance and non-substance-related addictive behaviors. Therefore, it is possible to speculate that behavioral addictions could be considered as an expression of an individual underlying psychopathological fragility, rather than symptoms of excessive involvement in maladaptive activities *per se* (79).

Among young people, academic or educational impairment owing to missed classes, falling behind on work, and lower grades was associated with binge drinking (80). Our study revealed that the frequency of subjects reporting failure/poor school performance was almost two times higher among students with a lifetime substance consumption. Similarly, as recently described in other countries (81, 82), problematic Internet use and gambling were more common in students who reported failure/poor school performance. Therefore, it is possible to state that both substance and non-substance addictive behaviors might negatively influence the educational path of adolescents.

To the best of our knowledge, this is the first study investigating the association of substance- and non-substance-related addictive behaviors with distinct psychopathological dimensions and perceived poor academic performance in a sample of Italian adolescents.

However, this study presents few limitations, such as the relatively small sample size, the predominance of females, and the cross-sectional nature that precluded the ability to identify the chronological order of the onset of different addictive behaviors.

Given the negative impact on adolescents’ quality of life, and the increased risk for violent/aggressive behaviors (83) as well as for addictive and other psychiatric disorders in adulthood (84), early recognition of alcohol/substance use, binge drinking, and addictive behaviors becomes crucial. Further studies are necessary to better define psychopathological correlates of addictive behaviors in adolescents, to clarify, prevent, and curb this phenomenon.

## Ethics Statement

This study was carried out in accordance with the recommendations of Good Clinical Practice guidelines and the Declaration of Helsinki (1964) and subsequent revisions. All subjects (or parents/tutors, if they were underage) gave written informed consent. The protocol was approved by the Ethics Committee of Università Cattolica del Sacro Cuore, Rome, Italy.

## Author Contributions

MN and LJ were primarily responsible for study design and contributed to data interpretation and article writing. AS and PG performed data collection and contributed to data interpretation. VF and DT were involved in data entry and database management. AC performed statistical analysis. GM and FD-G were involved in data interpretation. VF, LM, and IP contributed to data interpretation and article writing. All authors personally revised and approved the final version of the manuscript.

## Conflict of Interest Statement

The authors declare that the research was conducted in the absence of any commercial or financial relationships that could be construed as a potential conflict of interest.
